# Prevalence of opioid-induced adverse events across opioids commonly used for analgesic treatment in Japan: a multicenter prospective longitudinal study

**DOI:** 10.1007/s00520-023-08099-2

**Published:** 2023-10-16

**Authors:** Yusuke Hiratsuka, Keita Tagami, Akira Inoue, Mamiko Sato, Yasufumi Matsuda, Kazuhiro Kosugi, Emi Kubo, Maika Natsume, Hiroto Ishiki, Sayaka Arakawa, Masaki Shimizu, Naosuke Yokomichi, Shih-Wei Chiu, Mayu Shimoda, Hideyuki Hirayama, Kaoru Nishijima, Kota Ouchi, Tatsunori Shimoi, Tomoko Shigeno, Takuhiro Yamaguchi, Mitsunori Miyashita, Tatsuya Morita, Eriko Satomi

**Affiliations:** 1 Department of Palliative Medicine, Takeda General Hospital, Aizu Wakamatsu, Japan; 2 Department of Palliative Medicine, Tohoku University Graduate School of Medicine, Sendai, Japan; 3 Department of Palliative Medicine, National Cancer Center Hospital East, Kashiwa, Japan; 4 Department of Palliative Medicine, National Cancer Center Hospital, Tokyo, Japan; 5 Department of Palliative Care, Kyoto-Katsura Hospital, Kyoto, Japan; 6 Division of Palliative and Supportive Care, Seirei Mikatahara General Hospital, Hamamatsu, Japan; 7 Division of Biostatistics, Tohoku University Graduate School of Medicine, Sendai, Japan; 8 Department of Palliative Nursing, Tohoku University Graduate School of Medicine, Sendai, Japan; 9 Department of Palliative Care, Kyowakai Medical Corporation, Daini Kyoritsu Hospital, Kawanishi, Japan; 10 Department of Medical Oncology, Tohoku University Hospital, Sendai, Japan; 11 Department of Medical Oncology, National Cancer Center Hospital, Tokyo, Japan; 12 Research Association for Community Health, Hamamatsu, Japan

**Keywords:** Opioid, Adverse event, Advanced cancer, Specialized palliative care

## Abstract

**Purpose:**

Although opioids have been shown to be effective for cancer pain, opioid-induced adverse events (AEs) are common. To date, little is known about the differences in risks of AEs by opioid type. This study was performed to compare the prevalence of AEs across opioids commonly used for analgesic treatment in Japan.

**Methods:**

This study was conducted as a preplanned secondary analysis of a multicenter prospective longitudinal study of inpatients with cancer pain who received specialized palliative care for cancer pain relief. We assessed daily AEs until termination of follow-up. We rated the severity of AEs based on the Common Terminology Criteria for Adverse Events version 5.0. We computed adjusted odds ratios for each AE (constipation, nausea and vomiting, delirium, and drowsiness) with the following variables: opioid, age, sex, renal dysfunction, and primary cancer site.

**Results:**

In total, 465 patients were analyzed. Based on the descriptive analysis, the top four most commonly used opioids were included in the analysis: oxycodone, hydromorphone, fentanyl, and tramadol. With respect to the prevalence of AEs among all analyzed patients, delirium (*n* = 25, 6.3%) was the most frequent, followed by drowsiness (*n* = 21, 5.3%), nausea and vomiting (*n* = 19, 4.8%), and constipation (*n* = 28, 4.6%). The multivariate logistic analysis showed that no single opioid was identified as a statistically significant independent predictor of any AE.

**Conclusion:**

There was no significant difference in the prevalence of AEs among oxycodone, fentanyl, hydromorphone, and tramadol, which are commonly used for analgesic treatment in Japan.

## Introduction

Cancer pain has a significant impact on patients’ quality of life [1]. One study showed that the pain prevalence rate was 44.5% in all cancer stages [2]. Opioids are often prescribed for the treatment of cancer pain as recommended by international guidelines [3, 4]. Although opioids have been shown to be effective for cancer pain, opioid-induced adverse events (AEs) are common [5]. The most common AEs are constipation, nausea and vomiting, delirium, and drowsiness [6]. All opioids can cause constipation, and opioid treatment is often only one of multiple factors contributing to constipation. Opioids may cause nausea and vomiting through both central and peripheral mechanisms [7]. Mild cognitive impairment is common following the initiation of opioid therapy or an increase in dose [8]. Initiation of opioid treatment or significant dose escalation commonly induce drowsiness. Several factors predictive of AEs have been reported: administration route [9], aging [10], drug interactions [11], and opioid dose. However, little is known about the differences in risks of AEs by opioid type [12].

To date, no study has been conducted to investigate the prevalence of AEs among Japanese patients with advanced cancer who received specialized palliative care. We hypothesized that understanding the characteristics of AEs can improve the quality of analgesic treatment using opioids. This study was performed to compare the prevalence of AEs (constipation, nausea and vomiting, delirium, and drowsiness) across opioids commonly used for analgesic treatment in Japan.

## Methods

### Participants

This study was a preplanned secondary analysis of a multicenter prospective longitudinal study conducted in Japan from 1 November 2020 to 30 June 2021. Five specialized palliative care consultation teams and four palliative care units in tertiary care hospitals in Japan participated in the study. The original study assessed the time to achieve cancer pain management by specialized palliative care provided by certified palliative care physicians, advanced practice nurses, and multidisciplinary palliative care staff (psycho-oncology physicians, pharmacists, medical social workers, rehabilitation specialists, and dentists, among others). We defined cancer pain in this study as pain associated with cancer invasion and metastasis; the definition of cancer pain excluded pain related to anticancer treatments such as surgery, chemotherapy, and radiotherapy. In patients with multiple cancer pain, we focused on the cancer pain that was most severe and interfered with daily life. We enrolled consecutive eligible inpatients who received specialized palliative care. All observations were performed within the range of routine clinical practice. The inclusion criteria for the patients in the original study were 1) receipt of specialized palliative care, 2) cancer pain, and 3) requirement for further cancer pain management. In this study, we added the inclusion criteria: the patients who were used opioids for cancer pain management. We excluded patients 1) whose most severe pain was not cancer pain, 2) who were admitted for surgery, and 3) who could not evaluate their symptoms and therapeutic interventions because of a severe symptom burden, serious psychological distress, or cognitive disorders preventing us from evaluating their symptoms or treatment. All patients were followed up until they indicated that there was no need for further cancer pain management because the management was sufficient, they developed other types of cancer pain that required more intervention, or they were discharged without adequate pain management. Although the patients had been previously registered, they were re-registered each time if they required further cancer pain management because of worsening or new pain.

### Data collection

Palliative care physicians prospectively recorded the data collection sheets every day to determine whether the enrolled patients considered their cancer pain management successful and whether they needed further management. We collected the following patient data at enrollment (baseline): age, sex, primary cancer site, organ dysfunction (liver dysfunction: total bilirubin of > 1.5 mg/dL and renal dysfunction: estimated glomerular filtration rate of < 45 mL/min/1.73 m^2^), concomitant drugs (nonsteroidal anti-inflammatory drugs, acetaminophen, and analgesic aids), opioids, and daily morphine milligram equivalents. AEs were assessed every day until termination of follow-up.

### Measurements

We performed a daily assessment of the severity of constipation, delirium, drowsiness, nausea, and other AEs due to analgesics and analgesic adjuvants until termination of follow-up. We rated AEs from grade 1 to 5 based on the Common Terminology Criteria for Adverse Events version 5.0 [13]. A patient was considered to have an AE if the causal relationship between opioids and AEs was “possible,” “probable,” or “definite” and the grade was ≥ 1. When the same AEs occurred more than two times during the observation period, we only recorded the higher-grade events.

We calculated the dose of opioid at enrollment as daily morphine milligram equivalents based on previous studies [14, 15]: oral morphine 60 mg = intravenous (IV)/subcutaneous (SC) morphine 30 mg = oral oxycodone 40 mg = IV/SC oxycodone 30 mg = fentanyl patch 25 μg/h = IV/SC fentanyl 0.6 mg = oral tapentadol 200 mg = oral hydromorphone 12 mg = IV/SC hydromorphone 2.4 mg = oral tramadol 300 mg.

### Data analysis and statistics

First, we extracted opioid-using patients from all enrolled patients. We excluded patients who received analgesic treatment by only non-opioids, such as nonsteroidal anti-inflammatory drugs.

Second, descriptive analyses were performed to summarize the analyzed baseline patient characteristics.

Third, multivariate logistic analysis was performed to compute the adjusted odds ratio (OR) for each AE with the following variables: opioid (reference group = oxycodone), age (per 10 years), sex (reference group = male), renal dysfunction (reference group = absent), and primary cancer site (digestive tract, lung, and others; reference group = others). As for opioid, we selected oxycodone because of the most frequency prescription in the analyzed patients. Opioids prescribed to less than 30 patients were excluded from the analysis because they would destabilize the logistic model. Potential confounding variables were identified based on previous studies [9–11].

All analyses were performed using JMP version 16 for Windows (SAS Institute, Cary, NC). A *p* value between 0.01 and 0.05 was considered to be a weak indication of the finding, and a *p* value of < 0.01 was considered to be a clear indication of a finding.

### Ethics

The present study was conducted in accordance with the ethical standards of the Declaration of Helsinki and the ethical guidelines for medical and health research involving human subjects presented by the Ministry of Health, Labour and Welfare, Japan. This study was approved by the institutional review board of each participating institution and by the Independent Ethics Committee of Tohoku University School of Medicine (approval no. 2020–1-1085). Japanese law does not require individual informed consent from participants in a noninvasive observational trial such as the present study. Therefore, we used an opt-out method rather than obtaining written or verbal informed consent. All patients could receive information on the study through the instructions posted on the ward or institutional website and had the opportunity to decline participation.

## Results

### Patient characteristics

In total, 522 patients were enrolled during the study period. Fifty-seven patients were excluded because of no opioid use. Thus, we analyzed 465 patients, including 265 (57.7%) men and 194 (42.2%) women (mean ± standard deviation age, 62.6 ± 13.8 years). Among the opioids, oxycodone (*n* = 189, 40.9%) was the most commonly used, followed by hydromorphone (*n* = 89, 19.3%), fentanyl (*n* = 80, 17.3%), tramadol (*n* = 52, 11.3%), tapentadol (*n* = 29, 6.3%), and morphine (*n* = 20, 4.3%). The median observation duration was 3.0 days (range, 1.0–7.0 days). The patients’ characteristics are shown in Table 1.

**Table 1:** Patient characteristics

Characteristics	Total (*n* = 465)	Tramadol (*n* = 51)	Oxycodone (*n* = 180)	Hydromorphone (*n* = 84)	Fentanyl (*n* = 80)	Tapentadol (*n* = 26)	Morphine (n-20)	Others (*n* = 24)
Age, years (missing, *n* = 18)	62.6 ± 13.8	64.6 ± 11.6	63.4 ± 11.6	63.5 ± 15.3	62.9 ± 15.1	58.2 ± 15.6	57.1 ± 15.5	58.1 ± 14.3
Sex								
Male Female Missing	265 (57.0) 194 (41.7) 6 (1.3)	37 (72.6) 14 (27.5) 0 (0.0)	113 (62.8) 64 (35.6) 3 (1.7)	40 (47.6) 44 (52.4) 0 (0.0)	40 (50.0) 39 (48.8) 1 (1.3)	15 (57.7) 10 (38.5) 1 (3.8)	11 (55.0) 9 (45.0) 0	9 (37.5) 14 (41.7) 1 (4.2)
Primary cancer site								
Lung Digestive tract Hepatobiliary/pancreas Breast Urological system Head/neck Gynecological system Hematological system Soft tissue Others Missing	109 (23.6) 104 (22.5) 45 (9.7) 13 (2.8) 34 (7.4) 32 (6.9) 39 (8.4) 25 (5.4) 27 (5.8) 35 (7.6) 2 (0.4)	9 (18.0) 8 (16.0) 3 (6.0) 0 (0.0) 11 (22.0) 6 (12.0) 5 (10.0) 2 (4.0) 3 (6.0) 4 (8.0) 0 (0.0)	58 (32.2) 42 (23.3) 26 (14.4) 6 (3.3) 14 (7.8) 5 (2.8) 11 (6.1) 0 (0.0) 5 (2.8) 12 (6.7) 1 (0.6)	20 (23.8) 11 (13.1) 9 (10.7) 2 (2.4) 4 (4.8) 1 (1.2) 10 (11.9) 11 (13.1) 8 (9.5) 8 (9.5) 0 (0.0)	5 (6.3) 29 (36.3) 3 (3.8) 2 (2.5) 3 (3.8) 12 (15.0) 8 (10.0) 8 (10.0) 3 (3.8) 7 (8.8) 0 (0.0)	8 (30.8) 3 (11.5) 2 (7.7) 0 (0.0) 1 (3.8) 1 (3.8) 2 (7.7) 3 (11.5) 5 (19.2) 1 (3.8) 0 (0.0)	2 (10.0) 5 (25.0) 0 (0.0) 1 (5.0) 0 (0.0) 6 (30.0) 1 (5.0) 1 (5.0) 2 (10.0) 2 (10.0) 0 (0.0)	7 (29.2) 6 (25.0) 2 (8.3) 2 (8.3) 1 (4.2) 1 (4.2) 2 (8.3) 0 1 (4.2) 1 (4.2) 1 (4.2)
Organ dysfunction								
Liver dysfunction								
Present Absent Missing	24 (5.2) 429 (92.3) 12 (2.6)	2 (3.9) 48 (94.1) 1 (2.0)	11 (6.1) 165 (91.7) 4 (2.2)	3 (3.6) 80 (95.2) 1 (1.2)	6 (7.5) 71 (88.8) 3 (3.8)	1 (3.8) 25 (96.2) 0 (0.0)	0 19 (95.0) 1 (5.0)	1 (4.2) 21 (87.5) 2 (8.3)
Renal dysfunction								
Present Absent Missing	48 (10.3) 405 (87.1) 12 (2.6)	8 (15.7) 42 (82.4) 1 (2.0)	14 (7.8) 162 (90.0) 4 (2.2)	8 (9.5) 75 (89.3) 1 (1.2)	11 (13.8) 66 (82.5) 3 (3.8)	3 (11.5) 23 (88.5) 0 (0.0)	1 (5.0) 18 (90.0) 1 (5.0)	3 (12.5) 19 (79.2) 2 (8.3)
Concomitant drugs								
Nonsteroidal anti-inflammatory drugs Acetaminophen Analgesic aids	226 (48.6) 231 (49.7) 144 (31.0)	17 (33.3) 28 (54.9) 16 (31.4)	113 (62.8) 97 (53.9) 48 (26.7)	38 (45.2) 44 (52.4) 32 (38.1)	22 (27.5) 31 (38.8) 17 (21.3)	13 (50.0) 6 (23.1) 13 (50.0)	8 (40.0) 14 (70.0) 6 (30.0)	15 (62.5) 11 (45.8) 12 (50.0)
Daily MME, mg (missing, *n* = 71)	74.2 ± 94.9	30.6 ± 15.1	85.2 ± 109.6	74.7 ± 82.1	47.4 ± 48.8	74.6 ± 67.1	73.5 ± 60.9	197.2 ± 171.9
Observation time, days	3.0 (1.0–7.0)	3.0 (1.0–7.0)	3.0 (1.0–7.0)	3.0 (1.0–7.0)	2.0 (1.0–7.0)	3.0 (1.0–7.0)	3.0 (1.0–7.0)	3.0 (1.0–7.0)

Data are presented as mean ± standard deviation, *n* (%), or median (range)

*MME* morphine milligram equivalents

### Adjusted ORs for AEs by opioids

The OR for each AE is shown in Table 2. Based on the descriptive analysis for opioid prescription, the top four most commonly used opioids were included in the analysis: oxycodone, hydromorphone, fentanyl, and tramadol. With respect to the prevalence of AEs for all opioids, delirium (*n* = 27, 5.8%) was the most frequent, followed by drowsiness (*n* = 23, 4.9%), nausea and vomiting (*n* = 22, 4.7%), and constipation (*n* = 20, 4.3%). With respect to the prevalence of AEs for the four analyzed opioids, delirium (*n* = 25, 6.3%) was the most frequent, followed by drowsiness (*n* = 21, 5.3%), nausea and vomiting (*n* = 19, 4.8%), and constipation (*n* = 28, 4.6%). After controlling for age (per 10 years), sex, renal dysfunction, and primary cancer site (digestive tract, lung, and others), no single opioid was identified as a statistically significant independent predictor of any AE. However, the following trends were shown: the risk of constipation was decreased with fentanyl (adjusted OR, 0.15; 95% confidence interval [CI], 0.02–1.23; *p* = 0.08) compared with oxycodone, and the risk of nausea and vomiting was increased with tramadol (adjusted OR, 3.03; 95% CI, 0.87–10.57; *p* = 0.08) compared with oxycodone. Additionally, the risk of constipation was clearly increased in patients with renal dysfunction (adjusted OR, 5.42; 95% CI, 1.61–18.22; *p* < 0.01).

**Table 2:** Multivariate analysis results: identification of factors associated with adverse effects

Variable	Constipation (*n* = 18)					Nausea and vomiting (*n* = 19)					Delirium (*n* = 25)					Drowsiness (*n* = 21)				
	*n* (%)	OR	aOR	95%CI	*p* value	*n* (%)	OR	aOR	95%CI	*p* value	*n* (%)	OR	aOR	95%CI	*p* value	*n* (%)	OR	aOR	95%CI	*p* value
Opioid																				
Tramadol	5 (9.8)	1.85	1.39	0.38–	0.62	5 (9.8)	1.85	3.03	0.87–10.57	0.08	4 (7.8)	0.94	0.67	0.17–2.68	0.58	2 (3.9)	0.57	0.55	0.11–2.71	0.47
Hydromorphone	2 (2.4)	0.41	0.37	0.08–1.79	0.22	2 (2.4)	0.41	0.54	0.11–2.68	0.45	4 (4.8)	0.55	0.66	0.20–2.19	0.50	3 (3.6)	0.52	0.58	0.15–2.19	0.42
Fentanyl	1 (1.3)	0.22	0.15	0.02–1.23	0.08	2 (10.5)	0.44	0.44	0.09–2.22	0.32	2 (2.5)	0.28	0.36	0.07–1.73	0.20	4 (5.0)	0.74	0.85	0.25–2.94	0.80
Oxycodone	10 (5.6)	ref	ref			10 (5.6)	ref	ref			15 (8.3)	ref	ref			12 (6.7)	ref	ref		
Age (per 10 years)	-	0.80	0.78	0.54–1.13	0.19	-	1.01	0.96	0.66–1.40	0.83	-	1.39	1.32	0.90–1.94	0.15	-	0.94	0.90	0.64–1.27	0.57
Sex																				
Female	9 (5.6)	1.39	2.08	0.74–5.81	0.16	11 (6.8)	1.68	2.75	0.98–7.77	0.06	5 (3.1)	0.37	0.44	0.16–1.26	0.13	5 (3.1)	0.36	0.47	0.17–1.35	0.16
Male	9 (3.9)	ref	ref			8 (3.5)	ref	ref			20 (8.7)	ref	ref			16 (7.0)	ref	ref		
Renal dysfunction	5 (12.2)	3.25	5.42	1.61–18.22	< 0.01	3 (7.3)	1.43	2.38	0.60–9.43	0.22	4 (9.8)	2.13	2.16	0.65–7.20	0.21	4 (9.8)	1.95	2.59	0.78–8.64	0.12
Primary cancer site																				
Digestive tract	5 (5.7)	1.09	1.51	0.45–4.98	0.50	7 (7.8)	2.22	3.06	0.96–9.72	0.06	4 (4.4)	0.67	0.92	0.27–3.13	0.90	5 (5.6)	1.21	1.06	0.34–3.31	0.92
Lung	2 (2.2)	0.61	0.58	0.12–2.94	0.51	4 (4.4)	1.28	1.32	0.31–5.64	0.71	9 (9.8)	1.51	1.58	0.57–4.43	0.38	5 (5.4)	0.95	1.21	0.37–3.96	0.75
Others	10 (4.7)	ref	ref			8 (3.8)	ref	ref			12 (5.7)	ref	ref			11 (5.2)	ref	ref		

*OR* odds ratio, *aOR* adjusted odds ratio, *CI* confidence interval, *ref* reference group

## Discussion

The current study showed that the prevalence of AEs was relatively low in the participating patients with advanced cancer. The multivariate logistic analysis showed that no single opioid was a statistically significant independent predictor of any AE.

Among the analyzed participants, 4.6% to 6.3% showed any AEs during the observation period. A Cochrane review reported that the prevalence of AEs was 17% to 30% for constipation, 14% to 23% for nausea, and 7% to 15% for vomiting [16]. A systematic review showed that the prevalence of AEs was 11% to 38% for constipation, 5% to 50% for nausea, 4% to 50% for vomiting, 70% to 80% for confusion, and 3% to 50% for drowsiness [6]. Our results are lower than these previously reported prevalence rates, particularly constipation and delirium. Successful opioid therapy requires that the benefits of analgesia clearly outweigh AEs. An understanding of AEs and optimal management of AEs are therefore essential skills for specialized palliative care providers. In the current study, specialized palliative care was provided to all patients, suggesting that specialized palliative care intervention might reduce the prevalence of AEs. Additionally, a basic education program called the Palliative Care Emphasis Program on Symptom Management and Assessment for Continuous Medical Education was developed for all physicians engaged in cancer care to provide primary palliative care in Japan [17]. Thus, primary palliative care provided by responsible physicians might also reduce the prevalence of AEs. However, our observation period was shorter than the observation period of most previous studies (at least 1 week) [16], which precluded the examination of long-term patterns in AEs. We terminated the observation after the achievement of personalized pain goal; thus, the observation period differed from patient to patient. This may have resulted in underreporting of AEs in the enrolled patients.

There was no significant difference in the prevalence of AEs among tramadol, oxycodone, hydromorphone, and fentanyl. These results are consistent with previous investigations in which there was very little evidence suggesting that any one opioid agonist has a substantially better AE profile than any other [12]. Our multivariate analysis showed the following trends: the risk of constipation was decreased with fentanyl compared with oxycodone, and the risk of nausea and vomiting was increased with tramadol compared with oxycodone despite large confidence intervals. One study suggested that tramadol showed more side effects than morphine [18]. A systematic review concluded that there is no evidence of a significant difference in AEs between oxycodone and morphine or hydromorphone [19, 20]. Some data indicate that constipation is less severe with transdermal fentanyl [8, 21, 22]. Thus, our results are consistent with previous studies. Additionally, we assumed that oral naldemedine might have effectively treated constipation and nausea and vomiting in the analyzed patients [23, 24]. Although there was no data on concomitant naldemedine prescription in this study, the prescription rate for naldemedine might be lower for tramadol than for oxycodone, hydromorphone, and fentanyl.

Our study had a larger sample size than most prior studies [6, 16]. However, it also had several limitations. First, AEs were objectively evaluated by participating clinicians and we excluded the patients with a severe symptom burden, serious psychological distress, or cognitive disorders preventing us from evaluating their symptoms or treatment. Patients’ subjective evaluation might have changed the prevalence of AEs and the association between opioids and the prevalence of AEs. Second, we did not collect information about combination or symptomatic treatment such as antiemetics and laxatives, including naldemedine, after enrollment in the study. Considering that all participating patients received specialized palliative care interventions, the patients likely received combination or symptomatic treatment for opioid therapy. Such treatment could reduce the prevalence of AEs. However, we believe our results reflect real-world practice with specialized palliative care intervention. The results suggest that specialized palliative care has the potential to reduce the prevalence of AEs. Third, very few patients received morphine. In most previous studies, patients who received morphine served as the control group, and we acknowledge that there are resultant difficulties in interpreting the results of this study. Finally, the opioid dose (daily morphine milligram equivalents) was not included in the multivariate model as it could be an intermediate factor between opioid types and AEs. The descriptive analysis showed that opioid dose differed among opioid types. Studies limited to strong opioids are needed in the future.

## Conclusion

There was no significant difference in the prevalence of AEs among oxycodone, fentanyl, hydromorphone, and tramadol, which are commonly used for analgesic treatment in Japan.

## Data Availability

The data that support the findings of this study are available from the corresponding author, Yusuke Hiratsuka, upon reasonable request. All authors agree to provide data to the journal for review if needed.

## References

[1] Kroenke K, Theobald D, Wu J, Loza JK, Carpenter JS, Tu W (2010). The association of depression and pain with health-related quality of life, disability, and health care use in cancer patients. J Pain Symptom Manage.

[2] Snijders RAH, Brom L, Theunissen M (2023). Update on prevalence of pain in patients with cancer 2022: a systematic literature review and meta-analysis. Cancers (Basel).

[3] Caraceni A, Hanks G, Kaasa S (2012). Use of opioid analgesics in the treatment of cancer pain: evidence-based recommendations from the EAPC. Lancet Oncol.

[4] Swarm RA, Dans M (2018). NCCN frameworks for resource stratification of NCCN guidelines: adult cancer pain and palliative care. J Natl Compr Canc Netw.

[5] Corli O, Santucci C, Corsi N (2019). The burden of opioid adverse events and the influence on cancer patients’ symptomatology. J Pain Symptom Manage.

[6] Oosten AW, Oldenmenger WH, Mathijssen RH (2015). A systematic review of prospective studies reporting adverse events of commonly used opioids for cancer-related pain: a call for the use of standardized outcome measures. J Pain.

[7] Sande TA, Laird BJA, Fallon MT (2019). The management of opioid-induced nausea and vomiting in patients with cancer: a systematic review. J Palliat Med.

[8] Cherny N (2000). The management of cancer pain. CA Cancer J Clin.

[9] Müller-Lissner S, Bassotti G, Coffin B (2017). Opioid-induced constipation and bowel dysfunction: a clinical guideline. Pain Med.

[10] Pergolizzi J, Böger RH, Budd K (2008). Opioids and the management of chronic severe pain in the elderly: consensus statement of an International Expert Panel with focus on the six clinically most often used World Health Organization Step III opioids (buprenorphine, fentanyl, hydromorphone, methadone, morphine, oxycodone). Pain Pract.

[11] Kotlinska-Lemieszek A, Klepstad P, Haugen DF (2019). Clinically significant drug-drug interactions involving medications used for symptom control in patients with advanced malignant disease: a systematic review. J Pain Symptom Manage.

[12] Wiffen PJ, Wee B, Derry S (2017). Opioids for cancer pain - an overview of Cochrane reviews. Cochrane Database Syst Rev.

[13] Miyaji T, Iioka Y, Kuroda Y (2017). Japanese translation and linguistic validation of the US National Cancer Institute’s Patient-Reported Outcomes version of the Common Terminology Criteria for Adverse Events (PRO-CTCAE). J Patient Rep Outcomes.

[14] Knotkova H, Fine PG, Portenoy RK (2009). Opioid rotation: the science and the limitations of the equianalgesic dose table. J Pain Symptom Manage.

[15] Swarm RA, Paice JA, Anghelescu DL (2019). Adult cancer pain, version 3.2019, NCCN clinical practice guidelines in oncology. J Natl Compr Canc Netw.

[16] Wiffen PJ, Derry S, Moore RA (2014). Impact of morphine, fentanyl, oxycodone or codeine on patient consciousness, appetite and thirst when used to treat cancer pain. Cochrane Database Syst Rev.

[17] Yamamoto R, Kizawa Y, Nakazawa Y (2015). Outcome evaluation of the palliative care emphasis program on symptom management and assessment for continuous medical education: nationwide physician education project for primary palliative care in Japan. J Palliat Med.

[18] Wilder-Smith CH, Schimke J, Osterwalder B (1994). Oral tramadol, a μ-opioid agonist and monoamine reuptake-blocker, and morphine for strong cancer-related pain. Ann Oncol.

[19] King SJ, Reid C, Forbes K (2011). A systematic review of oxycodone in the management of cancer pain. Palliat Med.

[20] Pigni A, Brunelli C, Caraceni A (2011). The role of hydromorphone in cancer pain treatment: a systematic review. Palliat Med.

[21] Brenner DM, Stern E, Cash BD (2017). Opioid-related constipation in patients with non-cancer pain syndromes: a review of evidence-based therapies and justification for a change in nomenclature. Curr Gastroenterol Rep.

[22] Larkin PJ, Cherny NI, La Carpia D (2018). Diagnosis, assessment and management of constipation in advanced cancer: ESMO Clinical Practice Guidelines. Ann Oncol.

[23] Katakami N, Harada T, Murata T (2017). Randomized phase III and extension studies of naldemedine in patients with opioid-induced constipation and cancer. J Clin Oncol.

[24] Sato J, Tanaka R, Ishikawa H (2020). A preliminary study of the effect of naldemedine tosylate on opioid-induced nausea and vomiting. Support Care Cancer.

